# Genomic Regions Associated with Growth and Reproduction Traits in Pink-Eyed White Mink

**DOI:** 10.3390/genes15091142

**Published:** 2024-08-29

**Authors:** Hongyu Shi, Linling Liu, Peter Foged Larsen, Yu Ding, Tietao Zhang, Haihua Zhang, Zongyue Liu

**Affiliations:** 1 Jilin Provincial Key Laboratory for Molecular Biology of Special Economic Animals, Key Laboratory of Special Economic Animal Genetic Breeding and Reproduction, Ministry of Agriculture, Institute of Special Economic Animal and Plant Sciences, The Chinese Academy of Agricultural Sciences, Changchun 130112, China; shy20000314@126.com (H.S.); liulinling_600@126.com (L.L.); peter.foged.larsen@gmail.com (P.F.L.); zhangtietao@caas.cn (T.Z.); 2 Colleges of Animal Science, Hebei Normal University of Science and Technology, Qinhuangdao 066004, China; zhh83@126.com; 3 College of Animal Science, Jilin University, Changchun 130062, China; dingyu03@163.com

**Keywords:** mink, genome-wide association study, single-nucleotide polymorphism, total number born, number born alive, body weight

## Abstract

In mink breeding, balanced selection for growth and reproductive features is essential because these traits are contradictory. The variables of total number born (TNB), number born alive (NBA), and body weight (BW) are highly valuable in terms of their importance in mink production. A comprehensive understanding of the molecular mechanisms that drive these features could offer vital insights into their genetic compositions. In the present study, the single-nucleotide polymorphism (SNP) genotypes of 219 minks were obtained via double digest restriction-site associated DNA sequencing (ddRAD-seq). Following several rounds of screening, about 2,415,121 high-quality SNPs were selected for a genome-wide association study (GWAS). The GWAS was used to determine BW and reproductive traits in pink-eyed white mink. It was suggested that *SLC26A36*, *STXBP5L*, and *RPS 29* serve as potential genes for the total number of kits born (TNB), while *FSCB*, *PDPN*, *NKX 2-1*, *NFKB 1*, *NFKBIA*, and *GABBR1* are key genes for the number born alive (NBA). Moreover, *RTTN*, *PRPF31*, *MACROD1*, and *KYAT1* are possible BW genes based on association results and available functional data from gene and mammalian phenotype databases. These results offer essential information about the variety of mink and theoretical principles for applying mink breeds.

## 1. Introduction

Future economic advantages could be substantial if the genetic architecture behind conflicting features in mink breeding is unraveled. Within mink breeding, there is a detrimental genetic relationship between the weights of breeding animals and their reproductive characteristics. Hence, utilizing genetic markers to balance these characteristics in mink breeding initiatives is of utmost importance, specifically for white mink. The white color type is an essential factor in the fur business and breeding production due to its beneficial characteristics, including rapid growth, feed consumption, high fertility, and the option to be dyed any desired color [1]. The white color types, like Hedlund (hh) and Albino (cc), are recessive compared with wild-type mink. Mink that are homozygous for the h/h genotype show a complete absence of pigmentation, resulting in white coat color, deafness, and black eyes, whereas heterozygous individuals (h/+) display a piebald pattern [2]. The regal white (albino) phenotype in American mink is similar to the Hedlund white (Hedlund) type, but the eyes are pink, so it is also called pink-eyed white [3]. In 1937, M. Pirt in Winnipeg obtained a male mink with albinism, while in 1943, Lillie Herper in the US successfully bred individuals with albinism [4]. Completely white mink were produced by combining albino mink (cc) with royal pastel (bb) or Nordic buff (tntn) mink. 

Genomic research is a fundamental biological discipline that has the potential to transform the development of products and the improvement of animal production. Additionally, sequence information is becoming increasingly crucial for understanding the biology of all organisms. The genome sequence of mink at the chromosome level has been compiled via next-generation sequencing (NGS) technology (PacBio, HiSeq, and Hi-C) [5]. To ascertain the genetic diversity and structure of six mink fur color phenotypes from individuals from two farms, the Affymetrix Mink 70K panel was developed [6]. A few studies have identified candidate genes for economic attributes in mink. Cai et al. [7] conducted a GWAS analysis of mink BW and behavior and ten traits associated with fur quality. Further, the potential areas of response to Aleutian disease virus (ADV) infection in American mink were revealed via genome scanning [8]. Nevertheless, the efficacy of the GWAS was significantly constrained by the limited number of SNPs and the absence of chromosome-level genome sequence data until 2022.

Therefore, the current study aimed to analyze the genetic makeup of a mink breeding population and identify specific genetic markers associated with economically significant traits in white mink. The current understanding of gene function was combined with the findings to propose potential candidate genes responsible for these characteristics. These findings are biologically significant and can be used as recommendations for marker-assisted selection in mink.

## 2. Materials and Methods

### 2.1. Ethics Concerns

Animal handling and sampling protocols adhered to the strict guidelines of care and ethical concerns set forth by the Principle of Laboratory Animal Care and the Animal Research Committee of the Institute of Special Animals and Plants Sciences, Chinese Academy of Agricultural Sciences (Approval #: ISAPSAEC-2022-62M).

### 2.2. Animals and Phenotypes

In the breeding mink farm of the Yichun Huilongwan Company (Yichun, China), the mink population was uniformly reared. From 2019 to 2020, the data of approximately 219 reproductive minks were collected on this farm, including comprehensive pedigrees. The animals were subjected to dietary restrictions during the reproductive season. The mink had unlimited access to a range of nutritionally balanced diets for the rest of the year. This study examined reproductive traits, specifically the total number of kits born (TNB) and the number of kits alive during weaning (NBA). The TNB was observed during the initial 2 or 3 days after birth. The NBA represented the count of kits that were still alive 40 to 45 days after birth. Body length and BW were considered growth traits. The individual BW (±0.1 g) and body length (±0.1 cm) were noted during the third evaluation in December. The weights of the minks were recorded in the morning before feeding. Blood samples were drawn from the feet of the minks and kept at −20 °C. The Easy Pure Blood Genomic DNA reagent was used to extract genomic DNA from whole blood samples. The extracted DNA was quantified via an ultraviolet spectrophotometer. The DNA concentration, quality and integrity were determined by using a Qubit Fluorometer (Invitrogen, Carlsbad, CA, USA) and a Nano Drop Spectrophotometer (Thermo Scientific, Waltham, MA, USA).

### 2.3. Library Construction and Variant Calling

A previous protocol with slight modification was followed to construct double digest RAD sequencing (ddRAD-seq) libraries [9]. All samples were sequenced using the Illumina NovaSeq platform at Shanghai Personal Biotechnology (Shanghai, China). The quality of sequencing reads in each sample was measured via the Fast QC software (v0.11.7). Low-quality reads were trimmed, and overlapping reads (ORs) (>11 nucleotides (nt)/default) were combined (collapsed). Based on the recent reference genome of the common black mink (https://www.ncbi.nlm.nih.gov/assembly/GCF_020171115.1/, (accessed on 29 September 2021)), retrieved on 16 December 2022, the high-quality reads were aligned via BWA-mem v 0.7.12 [10]. In GATK (V3.8), SNPs with high confidence were declared. Moreover, individuals with missing genotypes < 15% were removed from the dataset using VCFtools 35. The SNPs and indels were functionally annotated via the ANNOVAR2017 software (V2017-07-16) [11].

### 2.4. Population Genome Analysis and Relationship Analysis

The population was analyzed for population structure, which included principal component analysis (PCA) and kinship analysis, to detect whether it was stratified. The GCTA software V3.8 was implemented to conduct PCA. The PCA results could be included as a covariate in the GWAS analysis to mitigate false-positive findings. The ADMIXTURE software (V1.3.0) was employed to execute the genetic evaluation of the population structure. The state homologous sequence IBS (identical by state) function in PLINK V 1.9 was used to estimate pairwise kinship via genome-wide autosomal SNP information. 

### 2.5. GWAS

The linear mixed-effects model was implemented to conduct correlation analysis using molecular markers and trait phenotypes to identify potential markers or essential genes closely associated with the target traits. The EMMAX software (beta-7 March 2010) was used to depict the results in Manhattan and Q–Q plots [12]. The model was explained in the following manner: Y = Xb + Za + e. The equation includes a vector, b, representing fixed effects like sex, age, gestation duration, and relatedness. The incidence matrices, X and Z, communicate the effects to the outcome variable, Y. The vector labeled e represents the random residuals. Table 1 provides a comprehensive overview of the fixed effects and covariates used in the analyses. Further, the genomic inflation factor (lambda, λ) values are provided to evaluate any potential systematic bias or substructure within the dataset.

### 2.6. LD and Haplotype Block Analysis

The LD study was conducted using the r^2^ statistic, and the Pop LD decay software (v3.40) was employed to generate the r^2^ values between the SNPs. The LD Block Show software (v-1.40) was used to visualize haplotype blocks, gene sequences often inherited as whole blocks, and haplotype networks based on SNP information.

## 3. Results

### 3.1. Statistical Description

This study used a sample size of 219 individuals that had both reproductive records and production performance data. Table 2 displays the statistical description of the outcomes for the TNB, NBA, and BW phenotypes in all mink samples.

### 3.2. SNP and Indel Calling

**Figure 1:**
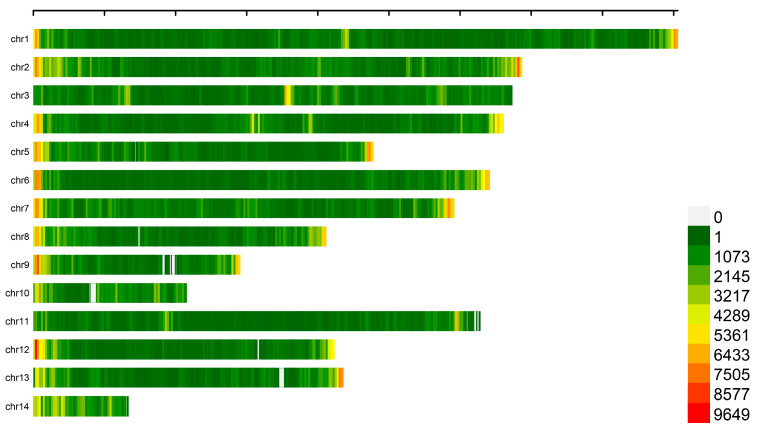
Distribution of variants within the autosomal and sex chromosomes of the mink genome.

**Figure 2:**
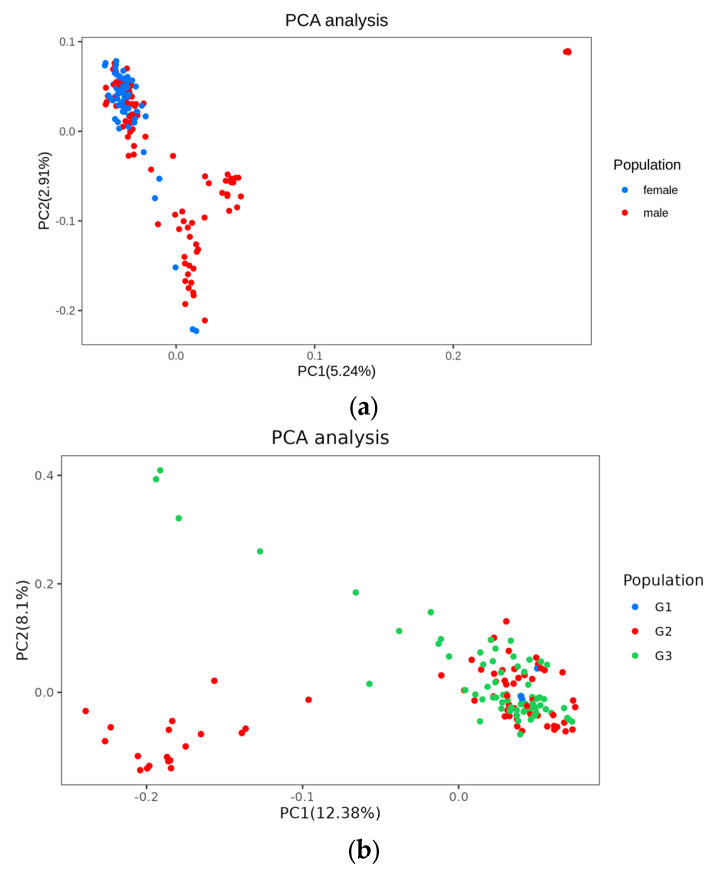
The principal component analysis of white mink populations by sex and generation. (**a**) PC1 = first principal component analyzed by sex; (**b**) PC2 = second principal component analyzed by generation. G1 was the parent generation, G2 was the offspring of G1, and G3 was the offspring of G2.

A total of 219 libraries were constructed using second-generation sequencing technology. The original data quality, respectively, 96.53 ≤ Q20 ≤ 97.9 and 91.74 ≤ Q30 ≤ 94.5, is illustrated in Supplementary Table S1. The GC content ranged from 47.99% to 54.95%, indicating the high quality of the sequence data. All filtered purified data were compared to the reference genome, resulting in a localization rate of 97.83% to 99.95%, as indicated in Supplementary Table S2. The findings of the sequence alignment showed that the localization rate satiated the necessary criterion for additional detection. The GATK software (v3.8) detected SNPs in every sample. After applying the MAF > 0.02 and max missing < 0.2 requirements, these SNPs were further filtered, yielding 2,415,121 high-quality SNPs for GWAS analysis. The number of SNPs was counted using 1Mb as the window, and their distribution on each chromosome is illustrated in Figure 1. The number of homozygous SNPs identified ranged from 278,729 to 396,752 and from 55,846 to 99,437 (Supplementary Table S3). The results of the SNP mutation analysis are displayed in Figure 2. The findings demonstrated that the primary SNP mutations were T: A → C: G and C: G → T: A. Table 3 reveals the SNP annotation results. The analysis revealed that 51.27% of the SNPs were found in the intergenic region, 42.22% of the SNPs were located in the intron region, 1.11% of the SNPs were situated in the upstream region, and 1.08% of the SNPs were clustered in the downstream region. Furthermore, the exon region contained 2.78% of the SNPs. In particular, 67,192 SNPs—including 30,498 synonymous polymorphisms, 34,431 non-synonymous polymorphisms, 30 stop-gain polymorphisms, and 1 stop-loss polymorphism—were found in the exon regions.

### 3.3. Population Structures

**Figure 3:**
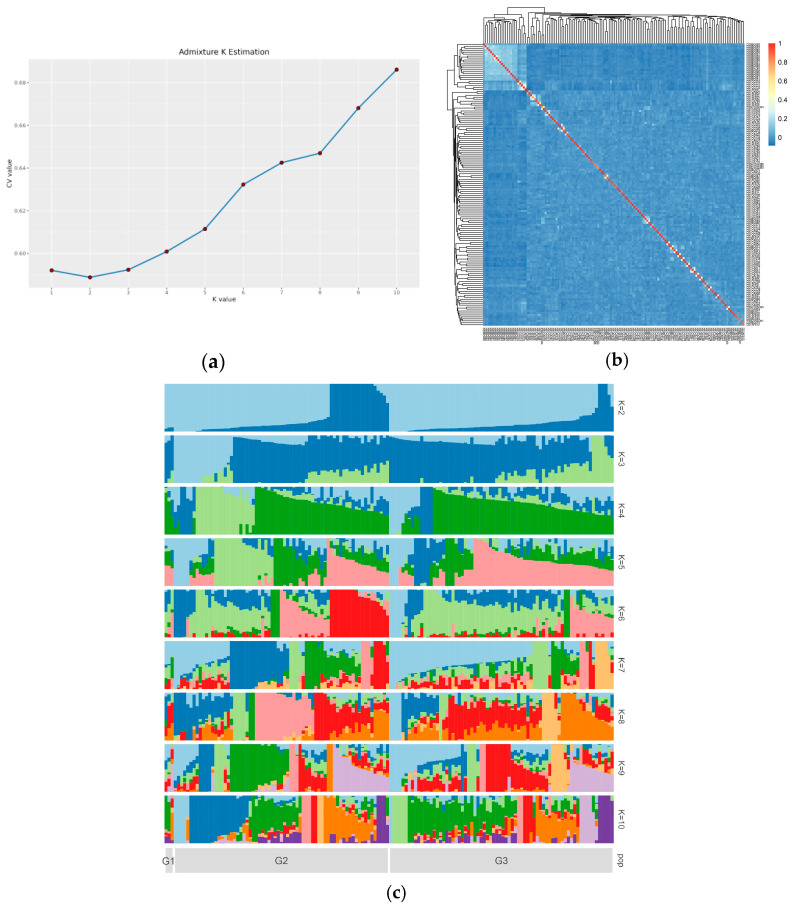
Population structure analysis of white mink. (**a**) Population structure of white mink with optimal K = 2; (**b**) kinship heat map; (**c**) population structure with K from 1 to 10. Each vertical line indicates one individual. Three generations are numbered at the bottom.

**Figure 4:**
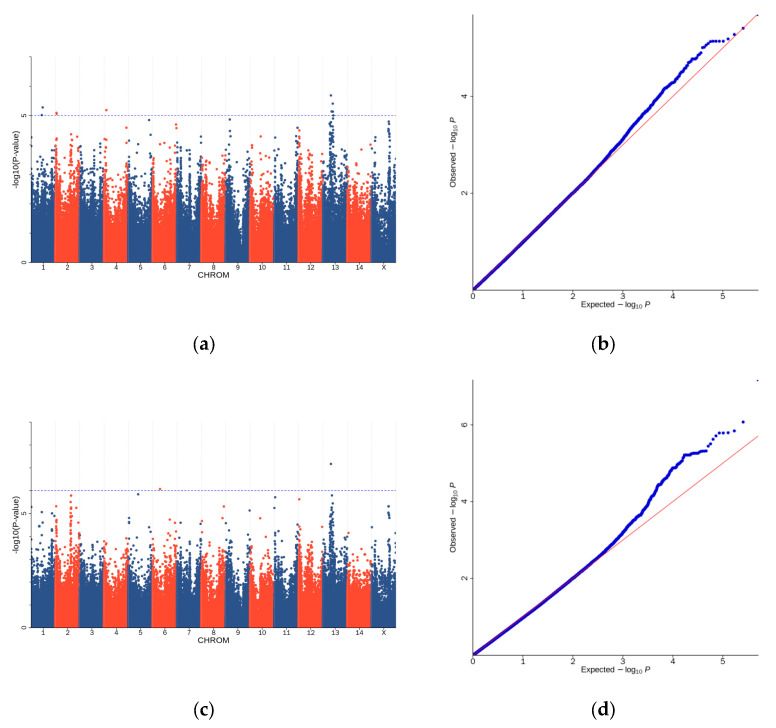

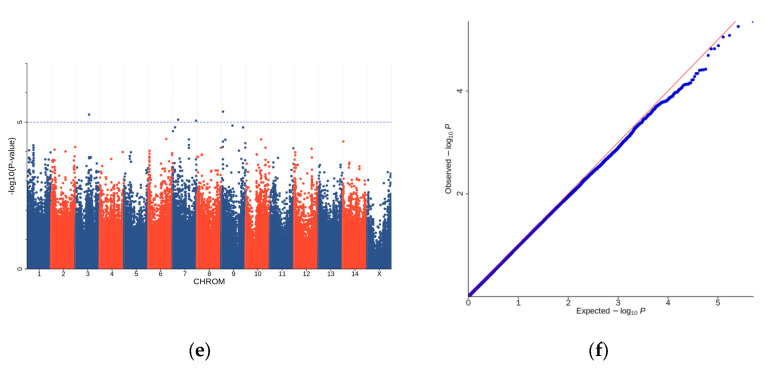
Manhattan and quantile–quantile (Q–Q) plots for GWAS analysis of the TNB, NBA, and BW. (**a**) Manhattan plot of GWAS results for TNB; (**b**) Q–Q plot of GWAS results for TNB; (**c**) Manhattan plot of GWAS results for NBA; (**d**) Q–Q plot of GWAS results for NBA; (**e**) Manhattan plot of GWAS results for BW; and (**f**) Q–Q plot of GWAS results for BW.

The population stratification of the white minks via PCA is highlighted in Figure 2. The lack of assignment of the mink population to distinct signaling clusters suggests that the reference population was not stratified. K = 2 was the optimal value for cross-validation, as illustrated in Figure 3a. The kinship estimation of all individuals indicated the effectiveness of sampling (Figure 3c). The kinship heat map, obtained from the IBS matrix (Figure 3b), displayed a population structure that aligned with the results of the PCA. Furthermore, there was no substantial indication of significant population stratification. The clusters were isolated from the G1, G2, and G3 generations of the white mink (Figure 4c). 

### 3.4. Significant SNPs and Genes from the GWAS

The association of mink traits was analyzed via EMMAX (KanHM et al., 2010) [12]. A total of 19 highly relevant SNPs were identified as being associated with TNB, NBA, and BW. The results of the GWAS showed that 2 SNPs were related to NBA, 13 SNPs were correlated with TNB, and 4 SNPs were associated with BW (Table 4). This study identified all potential genes located within a 100 kb region before and after the most prominent SNPs related to traits. The investigation identified all potential genes located near important SNPs associated with characteristics. The association analysis results were then annotated and can be found in the Additional Tables. Figure 4 also displays the Q–Q plots for these GWAS results.

### 3.5. LD and Haplotype Block Analysis

**Figure 5:**
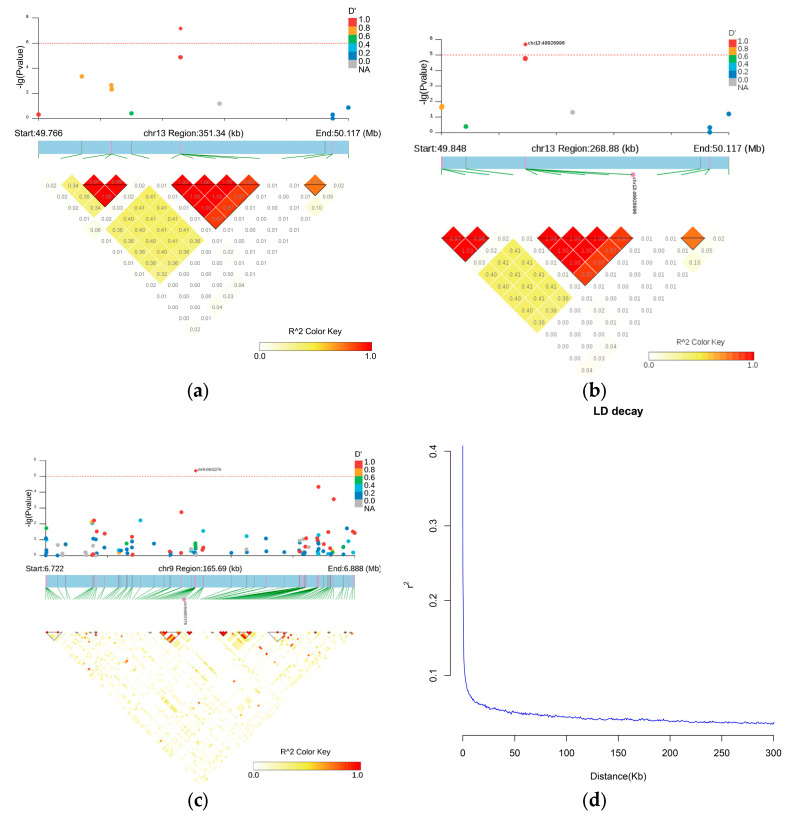
Haplotype plots: linkage disequilibrium blocks are determined in the regions, with markers in blocks shown in bold. (**a**): Haplotype plot of GWAS results for TNB; (**b**): haplotype plot of GWAS results for NBA; (**c**) haplotype plot of GWAS results for BW; and (**d**): LD in white mink population, with r^2^ values averaged throughout 0.5 Mb between physical distances of paired SNPs.

The LDBlockShow software (v-1.40) was used to obtain LD blocks based on the population SNP information. Figure 5 reveals the LD blocks for potential regions on SSC9 and SSC11, as predicted by the LD analysis results. In these regions, 49.766 to 50.117 Mb on SSC13, 49.848 to 50.117 Mb on SSC13, and 6.722 to 6.888 Mb on SSC9, three, one, and three LD blocks were identified. The LD attenuation analysis of the white mink revealed a rapid decline in LD as the distance grew, with an r^2^ value lowering to 0.1. The average distance between nearby markers was 50 kb (Figure 5d).

## 4. Discussion

Historical records indicate that the Jinlin white mink was first bred in China. In the 1980s, this species was crossed with the pink-eyed white mink from Denmark and the brown mink from the Soviet Union [13]. Although there has been some recent progress in the ability to reproduce, the characteristics of its fur quality still do not satisfy the market’s requirements. Between 2000 and 2020, Chinese mink farms imported a significant quantity of pink-eyed white, silver-blue, and brown minks from Denmark using over 50 airplanes. Pink-eyed white mink accounted for around 40% of the total number imported [14]. Imported mink have recently acclimated to the harsh weather conditions found in the Liaoning and Shandong provinces and other cold places like the Heilongjiang province. Most of the GWASs using brown mink primarily focused on growth traits, fur quality [7,8], and Aleutian disease [15], while a few investigated reproduction traits. This study was the first to use ddRAD-seq to conduct a GWAS of the BW and reproductive characteristics of pink-eyed white mink in China.

In this study, 2,415,121 SNPs were obtained from 219 animals from two generations via ddRAD seq. The number of SNPs was significantly higher than in previous reports, such as the diversity of 1,396,257 SNPs in five-color minks in China [16], a study of LD patterns in 52,714 SNPs [17], and a GWAS of fur quality and body size using SNPs analysis of 34,816 samples [7]. When 98.86% of clean readings were successfully located in the mink reference genome, SNP results were found. The value obtained closely aligns with the findings of a recent study on whole-genome sequencing of American mink [18], which reported an average similarity of 98.24%.

One of the key price factors in mink production is the size of the pelt, which is closely associated with the BW at the time of pelting [19,20]. Liu et al. [21] discovered that Hedlund white showed a faster growth rate, reached maturity earlier, and developed increased mature weight compared with brown, standard, and mahogany sapphire black. In this study, the average BW of male white mink was 3.14 kg, and that of females was 1.65 kg. According to Do et al. [17], male and female black mink reached their maximal BWs at 31 (3.10 ± 0.36 kg) and 19 (1.63 ± 0.20) weeks. In another study, Sørensen et al. [22] reported that the mature BW of brown mink was lower at 26 weeks of age. The mean weights for males and females with good feed efficiency were 2.76 kg and 1.36 kg, respectively. This study determined that the heritabilities of body weight (BW) and pelt length are 0.49 and 0.42, respectively. The genomic heritability estimates were slightly lower, precisely 0.48 and 0.44, respectively [7]. In this case, genotyping data were obtained using an RAD to conduct a GWAS on white mink. Based on this investigation, four SNPs associated with BW were found. In addition, the RTTN gene codes for basal body organization, cohesion, symmetry, centriole synthesis, and replication were found. This has recently been associated with the development of disorders characterized by microcephaly. Abnormal brain development pathways and dysfunctional protein mutations are the primary causes of *RTTN*-related neurological issues, which include microcephaly, congenital dwarfism, mental retardation, ophthalmic symptoms, and epilepsy [23]. ADP-ribosylation, or ADPr, is a modification of proteins and nucleic acids that regulates several functions shared by all living kingdoms. Numerous vital biological functions, including WNT signaling and DNA repair, are impacted by ADPr. Mono-ADP ribosylhydrolase 1 (MACROD1) appears to be primarily found in the mitochondria, suggesting that the skeletal muscles are where it is highly expressed [24]. The impact of *MACROD1* depletion on a skeletal muscle cell line was investigated by Žaja et al. [25]. ADP-ribosylhydrolase activity can be somewhat specialized and may be affected by specific cell types and stress. A genetic condition known as Prader–Willi syndrome (PWS) manifests itself as a variety of behavioral, cognitive, and physical symptoms [26]. The primary causes of excessive weight gain in PWS patients are dysregulated appetite control mechanisms and hyperphagic behavior [27]. The most prevalent syndromic form of life-threatening obesity is PWS, which is related to severe obesity [28]. Hyperphagia and an imbalanced appetite control mechanism are the primary factors contributing to the excessive weight gain observed in individuals with PWS [26,29]. Individuals with PWS and steatosis revealed elevated levels of the *KYAT1* gene compared with those without the condition [30].

The efficacy of mink reproduction is primarily measured by the size of the litter at birth and weaning. According to Gautason [31], increasing the number of pelted offspring/breeding females can substantially enhance the overall economic output of fur production systems. In Denmark, the average litter size of mink increased from 3.6 kits to 5.3 kits between the 1970s and the 2010s [32]. Beata Serema [33] reported that the number of white mink pups born was 6.14 and that of those alive was 5.69, which was lower than this study’s findings. The heritabilities (± SE) were found to be 0.07 ± 0.03 for the overall number of kits born, 0.07 ± 0.02 for the number of live kits at birth, and 0.09 ± 0.04 for the number of living kits at weaning [34]. Several candidate genes, such as *SLC25A36*, *PDPN*, *FSCB*, *NKX 2-1*, *NFKBIA*, *NFKB1*, and *GABBR1*, were identified in this study. These genes are potentially associated with TNB and NBA. *SLC25A36* belongs to the solute carrier (SLC) family, essential in maintaining mitochondrial biogenesis. Its depletion in mouse embryonic stem cells (mESCs) has been associated with mtDNA depletion and mitochondrial dysfunction [35]. In GWAS analysis [36], *SLC29A1* was discovered to be related to the number of stillbirths in sows. Protein kinase A (PKA)-induced tyrosine phosphorylation regulates the fibrous sheath CABYR binding protein (FSCB) during spermatozoa capacitation. The SUMOylation of two key proteins, ROPN1/ROPN1L, correlated with PKA/A kinase activity and spermatozoa motility, was inhibited by FSCB phosphorylation [37]. Paul R. Shorten [38] discovered that the expressions of TKDP1, PAG11, and PDPN played significant roles in mediating the impact of energy balance on embryo survival in cows experiencing partial embryo losses. These findings suggest potential signaling pathways that may explain how energy balance and progesterone affect the growth and survival of embryos. PKA-induced tyrosine phosphorylation regulates FSCB during spermatozoa capacitation. This phosphorylated FSCB showed a substantially higher affinity for ROPN1/ROPN1L than non-phosphorylated FSCB [37]. The *NKX2-1* gene encodes a transcription factor (TF) that regulates the function of thyroid-specific genes and has a role in morphogenesis. In Canadian Dairy Holstein cattle [39] and Sahiwal cattle [40], this gene was associated with calving to first service and characteristics of days open in GWASs. NF-kB protein, encoded by the NFKB1 gene, controls the apoptosis of male germ cells and the expression of genes during spermatogenesis, while NFKBIA encodes a critical inhibitor of NF-kB [41]. Tao Wang [42] discovered an association between the risk of idiopathic male infertility and two genetic polymorphisms of the *NFKBIA* gene in China. The GABA-B1 subunit of the G protein-coupled receptor is encoded by the gene *GABBR1*, which has been identified as a relevant potential gene. This component forms a heterodimer with the GABA-B2 monomer, resulting in the formation of the GABA-B receptor. Elise Beau Vangeel [43] demonstrated that pregnancy stress corresponds to fetal DNA methylation modifications, revealing *GABBR1* as one of the best possible genes responsible for pregnancy anxiety in neonates.

In this study, the average distance between adjacent markers was 50 kb. In line with these results, Karim et al. [15] also showed that the American mink’s average LD (r^2^) decreased to 20 kb, with at least 120,000 SNP markers and a mean distance of 51 kb between associated markers. For chromosomal regions with numerous relevant SNPs centered around the primary SNP, haplotype block analyses were performed to determine further potential regions related to TNB, NBA, and BW characteristics. There has never been a report on these three QTLs. Considering the reduced genomic heritability of reproductive features, increasing sample sizes and conducting precise mapping to identify associated traits are necessary.

## 5. Conclusions

The total number of kits born, kits born alive, and BW were substantially correlated with 2, 4, and 13 genomic regions, respectively. Several genes, including several previously unknown, were identified as potentially linked to the overall number of offspring produced, the proportion of offspring that survive birth, and birth weight. Before incorporating these findings into genetic selection for white mink, it is imperative to conduct additional validation using a more extensive dataset. In summary, these findings will be advantageous for future genomic research and efforts to improve the genetic characteristics of mink. 

## Figures and Tables

**Table 1:** Fixed effects and covariates were fitted in the model for three traits.

Trait	Fixed Effects						λ
	Sex	Age	Length of Gestation	PCA1	PCA2	PCA3	
TNB	√	√	√	√	√	√	0.940
NBA	√	√	√	√	√	√	0.961
BW	√	√		√	√	√	0.956

Note: λ is the genomic inflation factor. “√" means we fit this effect in the model.

**Table 2:** The statistical description of the phenotypic data.

Traits	Female			Male
	TNB	NBA	BW	BW
Mean ± SD	6.45 ± 2.08	5.17 ± 1.71	1.65 ± 0.28	3.14 ± 0.31
Max	11.00	9.00	2.2	3.70
Min	2.00	0.00	1.04	2.42

Note: TNB: total number of kits born; NBA: number born alive; BW: body weight; SD: standard deviation.

**Table 3:** SNP annotation result statistics.

Types	Total	
	Number	%
Exonic total	67,192	2.78
Synonymous	34,431	1.43
Non-synonymous	30,498	1.26
Stop-gain	777	0.03
Stop-loss	35	0
Unknown	1451	0.06
Splicing	440	0.02
ncRNA total	44	0
ncRNA_exonic	44	0
ncRNA_splicing	0	0
ncRNA_exonic; splicing	0	0
ncRNA_intronic	0	0
Intronic	1,019,723	42.22
Intergenic	1,238,143	51.27
UTR5	7589	0.31
UTR3	27,628	1.14
UTR5;UTR3	2	0
Upstream	26,832	1.11
Downstream	26,171	1.08
Upstream; downstream	1357	0.06
Total	2,415,121	100

Stop-gain: an SNP that leads to obtaining the termination codon; stop-loss: an SNP that causes the loss of termination codons; unknown: unknown functional sites caused by errors in the gene structure annotation database used for annotation; intronic: contains sub-regions; splicing: splicing junction of the 2 bp region; UTR: untranslated region; upstream: the region 1 kb upstream of the transcription start site; downstream: the region 1 kb downstream of the transcription termination site.

**Table 4:** Reproductive and growth trait candidate SNPs and genes.

Traits	Chr	Position	MAF	log10(P)	Genes
NBA	6	67,634,385	0.142	6.0725631	*STXBP5L*, *SLC25A36*
	13	49,926,996	0.1562	7.1691173	*RPS29*, *LOC122893428*
TNB	1	13,908,286	0.1114	5.0168059	*ATXN1* (intronic)
	1	15,071,975	0.0369	5.2790484	*GABBR1* (521753)
	2	9,564,047	0.3286	5.0929676	*PDPN* (intronic)
	2	12,429,330	0.1337	5.0591373	*ARHGEF10L* (intronic)
	4	21,260,036	0.1108	5.1866097	*SAMD12* (intronic)
	13	49,926,996	0.1562	5.6864521	*RPS29* (629,685), *LOC122893428* (1,047,595)
	13	54,658,193	0.1628	5.1395122	*FSCB* (771,634), *LRFN5* (892,149)
	13	54,658,194	0.1628	5.1395122	*FSCB* (771,635), *LRFN5* (1,892,148)
	13	54,658,196	0.1628	5.1395122	*FSCB* (771,637), *LRFN5* (1,892,146)
	13	54,658,197	0.1628	5.1395122	*FSCB* (771,638), *LRFN5* (1,892,145)
	13	61,338,532	0.0909	5.4076409	*NKX2-1* (61,961), *LOC122894326* (104,961)
	13	62,308,394	0.0966	5.1340934	*NFKBIA* (28,135), *PSMA6* (32,817)
	13	65,124,124	0.1733	5.0066897	*AKAP6* (intronic)
BW	3	13,116,054	0.3636	5.2561596	*RTTN* (intronic)
	7	45,758,732	0.05398	5.0827756	*PRPF31* (exonic)
	7	19,922,808	0.2861	5.0507150	*MACROD1* (intronic)
	9	6,802,276	0.05398	5.3573332	*KYAT1* (UTR3)

## Data Availability

The original contributions presented in the study are included in the article/Supplementary Material, further inquiries can be directed to the corresponding author.

## Supplementary Materials

The following supporting information can be downloaded at: https://www.mdpi.com/article/10.3390/genes15091142/s1. Tables S1–S3: This study performed a single-step GWAS of four traits in the white mink population. A total of 2, 4, and 13 genomic regions were significantly associated with the total number of kits born, the number of born alive, and Body weight. We detected several genes, some of which were novel, showing potential associations with the total number of kits born (SLC26A36, STXBP5L, RPS 29), the number of born alive (FSCB, PDPN, NKX 2-1, NFKB 1, NFKBIA, GABBR1), body weight (RTTN, PRPF31, MACROD1, KYAT 1). These results require validation using a larger dataset before their implementation in genomic selection among white mink. Overall, our findings will be useful for further genomic studies and genetic improvement programs in mink.
